# Advanced paramedics and nurses can deliver safe and effective pre‐hospital and in‐hospital emergency care: An integrative review

**DOI:** 10.1002/nop2.866

**Published:** 2021-05-06

**Authors:** Jörgen Jansson, Maria Larsson, Jan Nilsson

**Affiliations:** ^1^ Department of Health Sciences Faculty of Health, Science, and Technology Karlstad University Karlstad Sweden; ^2^ Faculty of Social and Health Sciences Inland Norway University of Applied Sciences Elverum Norway

## Abstract

**Aim:**

To explore and present an overview of scope of practice among registered nurses and paramedics with an advanced level of education in pre‐hospital and in‐hospital emergency care.

**Design:**

An integrative literature review.

**Method:**

Studies published between 2006 and 2018 were retrieved by searching the databases CINAHL, PubMed, Scopus and Web of Science. Studies were selected by three independent researchers, and data were synthesized using thematic analysis.

**Results:**

The 25 studies identified focused on in‐hospital (*n* = 15) and pre‐hospital emergency care (*n* = 10) and included 13 professional titles originated from seven countries. The thematic analysis disclosed four themes; “Versatile care,” “Safe care based on precision and accuracy,” “Autonomous performance within boundaries” and “Beneficial towards patients and society.” Advanced paramedics’ and advanced nurses’ services are characterized as safe, of high quality and of public benefit. Their services are being used in everyday practice as well as directed to certain categories of patients.

## 1 INTRODUCTION

In several countries, advanced paramedics (APs) and advanced nurses (ANs) are used in the areas of pre‐hospital and in‐hospital emergency care. In 2015 the International Council of Nurses found that up to 70 countries either have or plan to introduce advanced nursing practice roles (http://international.aanp.org/Home/FAQ). Also, professional titles vary as shown by Sheer and Wong (2008), who reviewed 69 advanced practice roles in 35 different countries. As there is no consensus, it is difficult to fully understand APs and ANs role and scope of practice (Gardner et al., 2007; Stasa et al., 2014). Pre‐hospital and in‐hospital emergency care have several challenges in common in providing care and in the utilization of appropriate competencies. Exploring existing research in this field might lead to new ideas and developments in policy making regarding optimized resource utilization and advanced practice roles. Terms relating to APs and ANs roles and scope of practice used in this study are presented along with their definitions in Table 1.

**TABLE 1:** List of terms and definitions

Terms	Definitions related to the study
*Scope of practice*	Scope of practice refers to the tasks and activities which an advanced paramedic or advanced nurse is legislatively permitted to undertake, and which are based on their education, training, experience and competence (Stasa et al., 2014).
*Paramedics with advanced level of education*	Advanced paramedics includes paramedics with specialist or advanced level of education. Education at specialist level could include a bachelors or master's degree (College of paramedics, 2018). Paramedic practitioners are also referred to as advanced paramedics. Advanced paramedics are normally engaged in pre‐hospital care settings.
*Nurses with advanced level of education*	Advanced nurses refer to registered nurses with specialist or advanced level of education. Education at specialist level could include a bachelors or master's degree. Nurse practitioners are also referred to as advanced nurses and they are presumed to hold master's degree (International Council of Nurses, 2016). Advanced nurses can be engaged in pre‐hospital and hospital care settings.
*Advanced practice*	Advanced practice refers to advanced paramedics or nurses who possesses the skills and capacities to undertake practice outside the basic scope of practice and thereby differ from basic practice through their level of specialisation, advancement and role expansion (Dowling et al., 2013). The International Council of Nurses defines advanced practice as: registered nurses who has acquired expert knowledge, complex decision‐making skills and clinical competence for expanded practice (https://international.aanp.org/Practice/APNRoles, 2018–11). Advanced clinical practice defines the role but also the responsibility and integration of evidence‐based practice, practice development, education, research, consultation and administration (Dowling et al., 2013; Jokiniemi et al., 2012; Mantzoukas & Watkinson, 2007).
*Specialist and advanced competence*	Refers to practice outside the scope of practice of paramedics or registered nurses. According to Begley et al., (2013), the degree of decision‐making and accountability separates specialist and advanced practitioners rather than the tasks undertaken. Specialist roles are common in acute care settings (Canadian Nurses Association, 2008).
*Nurse or Paramedic practitioners*	“Practitioner” roles have developed internationally as a strategy to meet increased health care demand, to reduce transports to ED, to compensate for the lack of physicians and to increase patient satisfaction (Tohira et al., 2014). The “practitioner” often works autonomously and performs extended scope of practices, that is assessments, treatments, diagnostic tests and prescription of medicine (International Council of Nurses, 2016; Tohira et al., 2014). Practitioner roles are common in primary care settings (Canadian Nurses Association, 2008).

### 1.1 Background

International studies have shown that nurses with higher competence are associated with improved quality of care and a reduced proportion of complications (Aiken et al., 2014). Regarding pre‐hospital care, there is evidence showing that paramedics with extended competence can autonomously assess and treat patients at the scene and thereby reduce the numbers of patients transported to emergency departments. However, whether higher competence among paramedics and nurses in pre‐hospital care lead to improved care quality is less investigated (Evans et al., 2013).

Emergency care practitioners require broad professional competence and an understanding of a variety of conditions as well as the specific competence required to perform specialized procedures (Wilson et al., 2015). Although the organization of pre‐hospital care differs between countries, two different models of care are described; the Franco‐German model and the Anglo‐American model (Al‐Shaqsi, 2010). The former is characterized by *bringing the hospital to the patient* where pre‐hospital physicians attend to the patient, administer treatment and make care decisions at the scene, while the latter is characterized by *bringing the patient to the hospital* and few patients being treated at the scene.

In the past two decades, both pre‐hospital and in‐hospital emergency care have experienced major changes such as increased patient numbers, including many patients with low priority conditions, which impact on emergency resources, patient satisfaction and patient safety (Hoot & Aronsky, 2008; Lowthian, Cameron, et al., 2011; Lowthian, Jolley, et al., 2011). Increased demands on pre‐hospital resources are interrelated with emergency department attendances and contribute to overcrowding as well as a strain on hospital systems (Lowthian, Cameron, et al., 2011).

Clinical protocols (Ebben et al., 2013) and triage systems have been developed over time to reduce individual variation in the assessment and treatment of patients and to improve quality of care in both pre‐hospital and in‐hospital emergency care (Farrohknia et al., 2011; Khorram‐Manesh et al., 2011; Robertson‐Steel, 2006). A feature relating to the triage of patients that appears to be increasing in importance and complexity is the balancing of efficient resource management and patient safety (Gardett et al., 2013).

In Swedish pre‐hospital care, clinical roles and scope of practice are, to a great extent, similar for both ANs and registered nurses (RNs). Despite the nurse's different levels of education, they are in general assumed to share the same responsibilities and there are no clear differences in the protocols and guidelines that govern pre‐hospital emergency care (Rantala et al., 2019; Wihlborg, 2018). By applying an international focus and exploring examples of utilization of APs and ANs competence, this study can contribute towards a deeper understanding of their scope of practice in pre‐hospital and in‐hospital emergency care. The aim of the present study was therefore to explore and present an overview of the scope of practice among paramedics and registered nurses with an advanced level of education in pre‐hospital and in‐hospital emergency care.

Research questions:

- What kind of interventions and procedures are performed by APs and ANs?
- Where, how and to which patients are the interventions and procedures performed?
- What is the outcome of the care and service performed by APs and ANs?

## 2 METHODS

### 2.1 Integrative review of the literature

An integrative review of the literature was chosen to integrate and synthesize results from former studies (Whittemore & Knafl, 2005). This design was chosen to enable a more comprehensive understanding of the heterogenous phenomena of interest, that is competence and scope of practice of APs and ANs in pre‐hospital and in‐hospital emergency care. The stages reported by Whittemore and Knafl (2005) guided the review and consist of: problem identification, literature search, data evaluation, data analysis and presentation. The fundamental principles of the Cochrane Collaboration and Preferred Reporting Items for Systematic Reviews and Meta Analyses (PRISMA) (Moher et al., 2009) also informed the review process.

### 2.2 Eligibility criteria

Primary peer‐reviewed research studies written in English and using quantitative, qualitative or mixed methods, with focus on paramedics and nurses with an advanced level of education were eligible for inclusion. Studies with a focus on pre‐hospital emergency care or in‐hospital emergency/emergency department care, including minor injury units, were included. Studies focusing on more than one setting were included if either pre‐hospital or in‐hospital emergency care context findings could be separated from other findings. Studies describing competence, roles and scope of practices, of APs and ANs (i.e. roles, functions, interventions, procedures, patient satisfaction, mortality and morbidity, safety and economic analysis) were included. Based on the fact that APs and ANs roles in pre‐hospital and in‐hospital emergency care settings have developed in the recent two decades and both systems are exposed to challenges related to effective competence utilization and increased patient demand (Cooper & Grant, 2009; Williams, 2017), only studies published 2006 or later were found to be eligible.

### 2.3 Search strategy

Primary searches were performed in Cochrane library, PubMed and Google Scholar.

Primary searches did not generate any published reviews covering the same field and populations. However, the primary searches contributed to further understanding of the complexity of the area of research. The search for the integrative review was performed in PubMed, CINAHL, Scopus and Web of Science, databases well suited for finding published peer‐reviewed “paramedic” and nursing research. The search strategy was based on a modified PICO structure and was discussed with a university librarian to ensure rigour.

The search strategy is displayed in Table 2 (minor alterations of search terms were made in databases).

**TABLE 2:** Search strategy

Search strategy: search terms related to modified PICO	
Population:	"Nurse Clinicians" OR "specialist ambulance nurse*" OR "Emergency nurse*" OR "Critical care nurse*" OR "emergency medical technicians" OR "Specialist nurse*" OR "Paramedic practitioner" OR "Emergency care practitioner" OR "Acute care nurse practitioner" OR "advanced paramedic" OR "Advanced nursing practice”
AND	
Setting	"Emergency service, hospital” OR "Pre‐hospital care" OR "Emergency medical services" OR "Emergency care"
AND	
Outcome	Competence* OR " Professional knowledge" OR "Nursing knowledge" OR "Nursing skills" OR "Clinical competence*" OR "Nurses role"

### 2.4 Search and selection of literature

**FIGURE 1:**
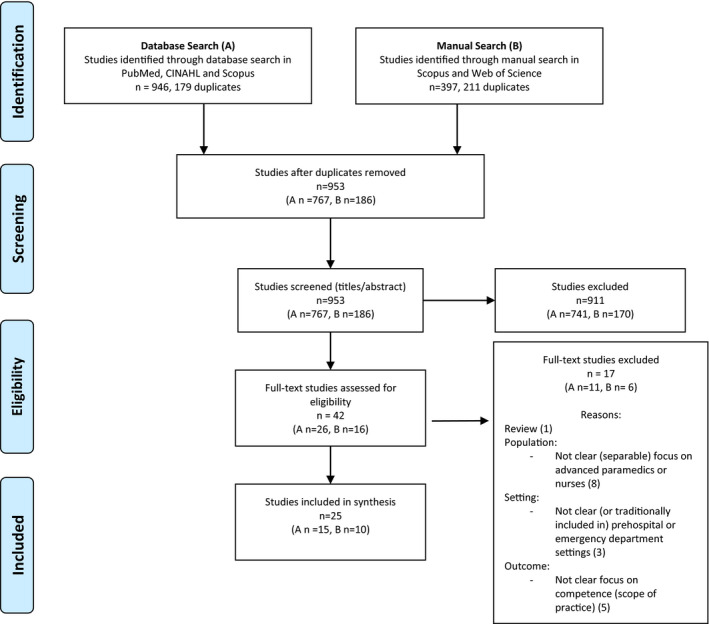
PRISMA 2009 flow diagram

The database searches, which was carried out in March 2017 and updated in September 2018, generated 946 records (Figure 1a), 179 were excluded as being duplicates leaving 767 records to be screened by title and abstract for relevance. Further, 741 records were rejected leaving 26 articles to be assessed in full text. A manual search in the databases Scopus and Web of Science was conducted in August 2017 and updated in November 2018 (Figure 1b). The same selection process was also used here. The manual search generated 397 studies: 211 were excluded for being duplicates leaving 186 records to be screened by title and abstract for relevance. Additionally, 170 records were rejected resulting in 16 articles to be assessed in full text. In total, 42 articles were assessed in full text; 17 did not meet the eligibility criteria leaving 25 studies to be included (Figure 1).

### 2.5 Data evaluation

A number of 25 studies were assessed using the Critical Appraisal Skills Program Tools (Public Health Resources Unit, 2006) for qualitative and quantitative studies modified by Nordström and Wilde‐Larsson and previously used by Sandsdalen et al., (2015). The modified critical appraisal tool was closely related to the original tool but also included a scoring system that facilitated categorizing the studies as being of either high, medium or low quality (Table 3). The assessments were based on total scores, as no areas or items were judged more significant than others. All steps of the selection process and quality assessment were performed by three independent researchers. All uncertainties or disagreements were discussed by the three authors until consensus was reached.

**TABLE 3:** Characteristics of included studies

Author/s (Year) Country	Study aim/s	Methods	Setting and Sample	Key findings	CASP level
1). Campo et al., (2008) United States	To describe the procedures and education being used by nurse practitioners (NP) in the emergency care setting (ECS) and the level of independence and confidence NPs report about performing these procedures.	A quantitative study. A cross‐sectional, descriptive design that used survey methodology.	Emergency department setting. NPs (*n* = 1799), response rate 23.5%, (*n* = 423).	NPs performed 71 procedures in the ECS and the majority did it independently and with confidence. Results also suggest that the higher the frequency of performing the procedures the higher the confidence and independence levels were. Examples of procedures performed include: fluorescein staining (*n* = 403), single layer closure, extremity or trunk (*n* = 399), nasal packing (*n* = 301), reduce fracture (*n* = 215), manage child CPR (*n* = 99) and balloon tamponade, oesophageal varices (*n* = 1).	High
2). Carlström & Fredén (2017) Sweden	To compare ambulance response times before and after the introduction of a single‐responder (SR) system and to explore job satisfaction of SRs.	A quantitative study. Statistical data related to response times were collected at the dispatch centre (Ambulink and Quickview). (Job satisfaction data were collected using a job satisfaction survey).	Pre‐hospital setting Measuring ambulance response times in a rural community during a 4‐month period in 2013.	The first SR system in Sweden (staffed with pre‐hospital emergency nurses) reduced the waiting time from 26 to 13 min (49% decrease) during the time of the study. Furthermore, it reduced ambulance transport in the project municipality by 35%.	High
3). Cummins et al., (2013) Ireland	To compare the diagnostic decisions of advanced paramedics (APs) with emergency medicine (EM) physicians, and to investigate if APs, as currently trained, can predict the requirement for hospital admission.	A quantitative study. The APs were asked to provide a clinical diagnosis for each patient and to predict if hospital admission was required. The data were then cross‐referenced with the working diagnosis of the receiving emergency physician and the hospital admission records.	Pre‐hospital setting. Emergency calls (*n* = 1,369)	APs’ diagnostic decisions concordance with the receiving emergency physician represents 70% (525/748) and is mirrored with 70% (604/859) correct hospital admission predictions. Concordance rates were extremely high in the categories of airway and ventilation, burns, paediatrics, and pregnancy and pre‐delivery emergencies (100%). Cases of poisoning and overdose (94%), childbirth and neonatal resuscitation (91%) and diabetic emergencies (89%) were also very well recognised. Areas of weak concordance included environmental emergencies (0%), allergies and anaphylaxis (25%), no abnormality detected (33%), and head and spinal injuries (43%).	High
4). Dixon et al., (2009) United Kingdom	To assess the cost effectiveness of the paramedic practitioner (PP) scheme compared with usual emergency care.	A quantitative study. A cluster randomized controlled trial of PP scheme compared with usual emergency care of older people. Resource use data were collected from routine sources, and from patient‐completed questionnaires for events up to 28 days. EQ−5D data were also collected at 28 days.	Pre‐hospital setting. Routine data, older people: intervention (*n* = 1,446) and control (*n* = 1,408). EQ−5D and QALY over 28 days: intervention (*n* = 646) and control (*n* = 568).	Whereas the intervention group received more PP contact time, it reduced the proportion of ED attendances (53.3% versus. 84.0%) and time spent in the ED (126.6 versus. 211.3 min). There was also some evidence of increased use of health services in the days following the incident for patients in the intervention group. Overall, total costs in the intervention group were £140 lower once routine data were considered (*p* =.63). When the costs and QALY were considered simultaneously, PP had a greater than 95% chance of being cost effective at £20 000 per QALY.	Medium
5). Griffin & McDevitt (2016) Ireland	To explore patients’ satisfaction and evaluate the quality of care provided by an advanced nurse practitioner (ANP) service in an emergency department.	A quantitative study. A prospective survey design was used for this study, which incorporated a self‐rated questionnaire. Patients were asked to rate their satisfaction on a survey. One open‐ended question was added to allow patients to comment on how the services could be improved.	Emergency department setting. A convenience sample of patients (*n* = 114), treated by the ANPs.	There was a high level of patient satisfaction associated with waiting time, pain management, advice given and communication. The median length of time from registration to the initial assessment by an ANP was 36 min. The median length of time from the initial assessment to either discharge or referral by the ANPs was 40 min. Overall, 78.6% of participants were seen and either discharged or referred within 60 min of the initial assessment by the ANPs. No significant correlation between waiting time and global satisfaction was revealed. No significant correlation between total time taken from the initial ANPs assessment to discharge or referral and total patient satisfaction was revealed. All patients with confirmed fractures had been correctly identified and appropriately managed by the ANPs.	Medium
6). Hui Lau et al., (2012) Australia	To compare assessment of suspected ankle and foot injuries using the Ottawa Ankle Rules (OAR) by nurse practitioners (NP) and emergency department (ED) doctors.	A quantitative study. A prospective, comparative, observational study.	Emergency department setting. A total of 174 patients were included in the study: 51 received NP and 123 received ED‐doctor care.	Assessed as requiring X‐ray assessment (NP: 78.4%, ED‐Dr: 88.6%; *p* =.081) and detection of significant fracture (NP: 17.6%, ED‐Dr: 22.8%; *p* =.453) were similar. ED‐based medical registrars were more likely to miss a fracture than NPs (NPs: 0%, ED‐based Registrars: 28.6%, *p* =.013). There were no significant differences in rates of OAR features for patients seen by NPs or ED‐doctors.	High
7). Jennings et al., (2015a) Australia	To evaluate the effectiveness of nurse practitioner (NP) service compared with standard emergency department medical care on clinical patient outcomes and key service indicators of waiting time, length of stay, unplanned representations, and left without being seen rates.	A quantitative study. A randomized controlled trial was conducted.	Emergency department setting. A total of 260 patients were enrolled in the study, 130 receiving NP care and 128 receiving standard emergency department (ED) care.	There were no significant differences between the two groups regarding waiting time, length of stay, numbers of patients who left, patient representations within 48 hr and the use of evidence‐based guidelines.	High
8). Jennings et al., (2015b) Australia	To evaluate quality of care delivered to patients presenting to the emergency department (ED) with pain and managed by emergency nurse practitioners (ENP).	A quantitative study. A retrospective chart review of consecutive patients with pain, managed by ENPs, was conducted. .	Emergency department setting. Consecutive patients with pain, managed by ENPs (*n* = 128), were included in the study.	Pain scores were documented in 67 (52.3%) patients. The median time to analgesia from presentation was 60.5 min, with 34 (26.6%) patients receiving analgesia within 30 min of presentation to hospital. There were 22 patients who received analgesia prior to assessment by an ENP. Among patients who received analgesia after assessment by an ENP, the median time to analgesia after assessment was 25 min, with 65 patients receiving analgesia within 30 min of assessment. The majority of patients assessed by nurse practitioners received analgesia within 30 min.	High
9). Jennings et al., (2013) Australia	To evaluate emergency nurse practitioner (ENP) service indicators as a measure of quality patient care.	A quantitative study. The study had a descriptive exploratory design.	Emergency department setting. Patients managed by ENPs (*n* = 5,212) were studied.	The median age of patients was 35 years and 61% of patients were male. The most common patient presentations managed by the ENP service were open wounds to wrist or hand (*n* = 547). Other common presentations included fracture of and unspecified parts of the wrist and hand (*n* = 292), surgical follow‐up care unspecified (*n* = 244) and sprain/strain of ankle unspecified (*n* = 202), respectively. Waiting time to be seen by the emergency nurse practitioner were 14 min, and length of stay for patients with a discharge disposition of home was 122 min. The most common discharge diagnosis was open wounds to hand/wrist. In total, there were 359 different discharge diagnoses described by the ENP service model. The most common discharge disposition was home (*n* = 4,509). The next most common discharge disposition was ward (*n* = 355) and short stay unit (*n* = 252). Patients were most commonly referred to their Local Medical Officer (*n* = 85%).	High
10). Magnusson et al., (2015) Sweden	To describe patient characteristics and assessment level made by the single responder (SR) nurse among patients assessed by the dispatcher as low priority and/or vague symptoms	A quantitative study. An exploratory descriptive study based on a consecutive retrospective review of patient notes.	Pre‐hospital setting. All patient notes (*n* = 529) where the SR (pre‐hospital emergency nurse) has been allocated during the first six months (June to November 2013) have been included in the study.	A total of 529 patients were assessed; 329 (62%) attended the emergency department (ED) or inpatient care (IC). Of these, 85 patients (26%) were assessed as high priority. Only 108 were assessed as in need of ambulance transport. ED/IC patients were significantly older. Two hundred (38%) patients stayed at the scene (SS) (*n* = 142) or were referred to PC (*n* = 58). Of the 200 SS/PC patients, 38 (19%) attended the ED within 72 hr with residual symptoms, 20 of whom were admitted to a ward. Nine patients (4% of 200 SS/PC patients) required inpatient treatment and 11 patients stayed overnight for observation. These results suggest a relatively high level of patient safety and the usefulness of an SR among patients assessed by the dispatcher as low priority.	Medium
11). Mason et al., (2006) United Kingdom	To describe the development of emergency care practitioner (ECP) schemes in 17 sites, identify criteria contributing to a successful operational framework and provide a preliminary estimate of costs.	A mixed‐method study The study included: 1). A quantitative study (a questionnaire to ECP project leads and routinely collected audit data from 2004) 2). Telephone interviews (semistructured, qualitative analysis) 3). Economic analysis	Pre‐hospital, emergency department and primary care setting. Pre‐hospital care patients (*n* = 2,724 patients), emergency department (ED) patients (*n* = 3,351) and patients in primary care (*n* = 2,500) were included. Telephone interviews (*n* = 12) with key personnel, three urban and three rural areas. Economic analysis was based on data from descriptive survey.	Of 17 sites, 14 (82.5%) responded to the questionnaire. Most of the operational ECPs had trained as paramedics in the ambulance service (77.4%). ECPs attended patients of all ages, but 79% (*n* = 6,803) were adults older than 16 years. The length of all ECP consultation (*n* = 7,673) was calculated and the median time was 25.0 min with a range of 1 min to 2 hr, pre‐hospital ECP 31 min (*n* = 2,490) and ED ECP 25 min median time (*n* = 2,998). Of the patients accessed via pre‐hospital service (*n* = 2,724, 32%), ECPs were able to assess, treat and discharge 43.5%. Of the patients who self presented to acute care settings (*n* = 3,351), the ECP:s referred 25% (*n* = 829) immediately to another acute care professional within the acute care setting itself. The ECP managed more than 58% of the cases without further referral. The economic analysis did not separate results between settings.	Medium
12). Mason et al., (2008) United Kingdom	To evaluate the safety of clinical decisions made by paramedic practitioners (PP) operating in the new service.	A quantitative study. The study was part of a cluster‐randomized controlled trial.	Pre‐hospital setting. Evaluation on care (*n* = 3,018), by PP (*n* = 1 549) and control (*n* = 1,469). A total of 157 records were analysed (clinical notes were missing in five cases).	Of the 2,025 patients included in this analysis, 219 (10.9%) went on to have an unplanned ED attendance within 7 days. Of these, 162 (74.0%) re‐presented with a condition related to their index episode. The independent raters (experienced senior ED clinicians) agreed on suboptimal care 83.4% of the time. There were 16 agreed upon episodes related to suboptimal care (0.80%). No significant differences were found between intervention and control groups in relation to re‐presentation at hospital within 7 days for a related condition or rates of assessed suboptimal care.	High
13). McConnell et al., (2013) United Kingdom	To determine the role and scope of emergency nurse practitioners (ENP) practice and establish the extent to which they could fulfil the criteria for an advanced nurse practitioner (ANP)	A quantitative study. A survey approach using a self‐reported questionnaire.	Emergency department and Minor Injury Unit setting. The sample included all ENPs (*n* = 42) working in Accident and Emergency Departments and Minor Injury Units in the region.	Investigation of role and scope of practice revealed a relatively homogenous group where the clinical aspect of the role dominated. The scope of practice was perceived to be influenced by internal factors such as competence; however, protocol use, referral rights and prescribing authority could be considered ways that nursing management and medical staff indirectly control the role. Examples of role features: order medications (90.5%) and X‐rays (97.6%), interpret X‐rays (92.9%), make diagnoses (95.2%), discharge (97.6%) and refer patients (97.6%). In all cases the clinical role dominated (mean 81.3%) with one ENP reporting spending 100% of the time in this role. Education and training (mean 5.7%) and expert resource (mean 4.8%) were the next highest followed by audit (mean 3.1%) leadership and management (mean 2.6%) and finally research (mean 2.5%).	High
14). McDevitt & Melby (2014) United Kingdom	To evaluate the quality of the emergency nurse practitioner (ENP) service.	A quantitative study. A descriptive study used a case‐note review and a survey design with one open‐ended exploratory question were conducted.	Rural urgent care centre (minor injuries) setting. A convenience sample was used and a total of 111 questionnaires were returned from patients treated by ENP (response rate 32%).	Despite comparatively low total length‐of‐stay times, most patients felt they had enough time to discuss things fully with the ENP. Although ENPs routinely impart injury advice, feedback from some patients suggests a need for the provision of more in‐depth information regarding their injury. The vast majority (97.3%) of patients felt that the quality of the ENP service was of high standard. Contrary to some other studies, the findings in this study indicate that patient satisfaction is not influenced by waiting time. ENPs in rural urgent care centres have the potential to deliver a safe and effective quality service that is reflected in high levels of patient satisfaction.	High
15). Norris & Melby (2006) United Kingdom	To explore the opinions of nurses and doctors working in emergency departments towards the development of an acute care nurse practitioner (ACNP) service in the UK…	A mixed‐method study The study had a descriptive, exploratory design and data were collected by: 1). Quantitative questionnaire to nurses and doctors about their opinions on the ACNP service and 2). Qualitative semistructured interviews from seven emergency departments and minor injury units.	Emergency department and minor injury units. Questionnaire (*n* = 98), nurses (*n* = 79) and doctors (*n* = 19). Interviews, nurses (*n* = 4) and doctors (*n* = 2).	While participants seemed comfortable with nurses undertaking traditional advanced skills such as suturing, reluctance was displayed with other advanced skills such as needle thoracocentesis. Three main themes were identified from the interviews: inter‐professional conflict, autonomy and the need for the ACNP. Doctors were reluctant to allow nurses to practise certain additional advanced skills and difficulties appear to be centred on the autonomy and other associated inter‐professional conflicts with the role of the ACNP. Nurses and doctors identified a need for the ACNP, but the blurring of boundaries between doctors and nurses can result in inter‐professional conflict unless this is addressed prior to the introduction of such advanced practitioners.	High
16). O Keeffe et al., (2011) United Kingdom	To evaluate the impact of emergency care practitioners (ECPs) on the patient care pathway for children presenting with minor conditions in unscheduled care settings	A quantitative study. The study was based on a quasi‐experimental multi‐site community intervention trial comparing ECPs with usual care providers.	Settings; 1). ECP working within GP‐led primary care out‐of‐hours service. 2). ECP‐led 24‐hr urgent care centre based in a community hospital. 3). ECP:s working in a minor injury unit. Paediatric acute episodes (*n* = 415 intervention and *n* = 748 control) were studied.	ECPs discharged significantly fewer patients than usual care providers. ECPs also referred more patients to hospital and primary care. ECPs are not as effective as usual health providers in discharging children after assessment of urgent healthcare problems. This has implications for the workload of other paediatric providers such as the emergency department. ECPs may be better targeted to settings and patients groups in which there is more evidence of their effectiveness in patient care pathways.	High
17). Rubenson et al., (2018) Sweden	To (1) explore pre‐hospital emergency care nurses’ (PECNs’) documented assessment and care of patients with head trauma and to (2) study gender differences in the documented care and interventions given by the PECNs.	A quantitative study. Pre‐hospital patient care records were analysed.	Pre‐hospital setting. A total of 2,750 patient records (patients with isolated head trauma)	The results showed that 25.2% of the patients were assessed according to all four core elements in the guidelines and 78.6% of the patients underwent at least one intervention by the PECNs. Nineteen of the total measured interventions (*n* = 2,161) had the character of being advanced (adv airway management *n* = 5, intravenous sedation *n* = 12 and vasoactive drugs *n* = 2). Male patients were assessed to a higher extent according to guidelines and were given higher transport priority while females were more often assessed for vital parameters and received significantly more analgesics. The assessment documented by the PECNs was not optimal concerning documentation using the Glasgow Coma Scale (35.2%), but the documented assessment of circulation and, especially, respiratory rate was high (77.2%).	High
18). Sandhu et al., (2009) United Kingdom	To carry out an in‐depth analysis of consultations made by emergency nurse practitioners (ENP) and to compare these to those of emergency department (ED) doctors and general practitioners (GP).	A quantitative study. Videotaped consultations were analysed and at the end of each consultation the doctor/ENP and the patient were asked to complete the Physician/ patient Satisfaction Questionnaire (PSQ).	Emergency department setting. A stratified sample was used. Patients presenting with "primary care" problems were allocated to senior house officers (SHOs, *n* = 10), specialist registrars/staff grades (*n* = 7), sessionally employed GPs (*n* = 12) or ENPs (*n* = 6). Six ENPs answered for 46 consultations and 29 doctors for 250 consultations.	ENPs and GPs focused more on patient education and counselling about the medical condition or therapeutic regimen than did ED doctors. There were no significant differences in consultation length. ENPs had higher levels of overall self‐satisfaction with their consultations than ED doctors. Patient satisfaction with information given in the consultation was significantly associated with the amount of talk relating to building a relationship.	Medium
19). Snaith & Hardy (2014) United Kingdom	To compare the radiographic interpretative accuracy and subsequent patient management plans of Emergency Nurse Practitioners (ENP) and medical practitioners within a single hospital trust with respect to musculoskeletal trauma.	A quantitative study. Patients were randomly allocated to the immediate or delayed reporting arm. ED clinicians were able to discuss the cases with examining radiographer and all of the assessment followed normal standard. Accuracy of radiographic interpretation and definitive diagnosis were based on a range of tests and assessments and not solely reliant on radiology report.	Emergency department setting. A convenience sample were made and patient records (*n* = 598), treated by either emergency nurse practitioners (ENP) or senior doctors, were analysed.	No significant difference in proportion of interpretive errors was evident between ENPs and medical staff.	High
20). Swain et al., (2012) New Zealand	To determine whether patients found the urgent community care (UCC) model of service both acceptable and effective, and to ascertain whether there was any difference in satisfaction with the care provided by the two groups of paramedics, standard ambulance service (EAS) or extended care paramedics (ECP).	A quantitative study. A patient satisfaction survey study was conducted. Patients attended by ECPs and by standard emergency ambulance service paramedics were interviewed (questionnaire).	Pre‐hospital setting. Patients attended by ECPs (*n* = 50) and by EAS (*n* = 50) were randomized by blind selection from a pool of numbers.	Patients were very satisfied with their experience of both groups of paramedics. Patients expressed a clear desire to be treated at home if possible. Of the 50 ECP‐treated patients, 11 were transferred directly to the ED. Only one clinical complication arose over the next 7 days in those treated in the community: a seizure in a patient with refractory epilepsy. The avoidance of unnecessary transfers to hospital is beneficial to patients, the ambulance service and the ED. This study demonstrates that patients are very satisfied with their assessment and treatment by ECPs, endorsing the proposal that the scheme should be extended across the Wellington Region, and perhaps New Zealand.	High
21). Thompson & Meskell (2012) Ireland	To provide empirical evidence for the outcomes of care of advanced nurse practitioners (ANPs) within the emergency department (ED). In addition, a comparison of ANPs with other medical clinicians working in the ED setting in relation to results of radiology investigations.	A quantitative study. A retrospective, comparative audit was undertaken in an ED of a general hospital. Data were retrieved manually from patient records, and statistical and descriptive analyses were undertaken.	Emergency department setting. Patient records (*n* = 964) were audited. Patients seen by profession: ANPs (*n* = 231) Consultant (*n* = 14) Registrars (*n* = 401) Senior house officer (*n* = 76) Non‐consultant hospital doctors (*n* = 76).	ANPs had equivalent if not better radiology diagnostic skills, awareness of pain management practices, and a greater impact on reducing patient waiting time than other grades of clinicians.	High
22). Van der Linden et al., (2010) Netherlands	To determine the incidence of missed injuries and inappropriately managed cases in patients with minor injuries and illnesses and to evaluate diagnostic accuracy of the emergency nurse practitioners (ENPs) compared with junior doctors/senior house officers (SHOs).	A quantitative study. A retrospective descriptive cohort study. Groups were compared regarding incidence and severity of missed injuries and inappropriately managed cases, waiting time and length of stay.	Emergency department setting. Patients treated by ENPs (*n* = 741) were compared with a random sample of patients treated by junior doctors/SHOs (*n* = 741).	ENPs showed high diagnostic accuracy, with 97.3% of the patients being correctly diagnosed and managed. Within the total group, 29 of the 1,482 patients (1.9%) had a missed injury or were inappropriately managed. No statistically significant difference was found between the ENP and physician groups in terms of missed injuries or inappropriate management, with 9 errors (1.2%) by junior doctors/SHOs and 20 errors (2.7%) by ENPs. There was no significant difference in waiting time for treatment by junior doctors/SHOs versus ENPs (20 min versus 19 min). The mean length of stay was significantly longer for junior doctors/SHOs (65 min for ENPs and 85 min for junior doctors/SHOs; *p* <.001; 95% confidence interval, 72.32–77.41).	Medium
23). Von Vopelius‐Feldt & Benger (2013) United Kingdom	To describe intubation success rates and complications, thereby contributing to the ongoing debate on the role and safety of this procedure.	A quantitative study. Data were collected retrospectively from 150 rapid sequence intubation (RSI) audit forms and electronic patient monitor printouts, between June 2008 and August 2011. Data were analysed using descripitive statistics.	Pre‐hospital setting. Pre‐hospital critical care teams, including critical care paramedics (CCP) (*n* = 8) and physicians (*n* = 18).	Critical care teams performed 150 RSIs between June 2008 and August 2011. The intubation success rate was 82, 91 and 97% for the first, second and third attempts, respectively. Successful intubation on the first attempt was achieved in 58 (85%) and 64 (78%) patients for physicians and CCPs, respectively. RSI complications included hypoxaemia (10.2%), hypotension (9.7%) and bradycardia (1.3%).	Medium
24). Von Vopelius‐Feldt & Benger (2014) United Kingdom	To provide the foundation for further discussion and research of critical care paramedics (CCP) practice, and to provide an overview of the current utilization and role of CCPs in England…	A quantitative study. An online national survey of all 12 regional National Health Services (NHS) in England was conducted. The survey consisted of a mixture of multiple choice and free text answers. Topics covered in the survey were CCP utilization, CCP training and competencies and CCP working patterns.	Pre‐hospital setting. CCPs (*n* = 90) at five different thrusts.	CCPs were used in five of twelve trusts. Reasons for not using CCPs were: insufficient financial means (5/7), insufficient scientific evidence (4/7), no clinical need (2/7) and no training scheme available (1/7). CCPs’ competencies vary between regions and include interventions like: fracture or joint reduction (4/5), sedation (4/5), RSI (0/5), surgical airway (4/5), postintubation sedation (2/5), non‐invasive ventilation (3/5), ultrasound (1/5), central lines (1/5), thoracotomy (1/5), chest drain (2/5). CCPs working patterns involves different settings: ambulance vehicle (1/5), rapid response vehicle (4/5), helicopter (5/5) as well as working alone or in a team: work alone (2/5), with paramedic/technician (2/5), with CCP (4/5) and with pre‐hospital physician (5/5).	Medium
25). Wolf et al., (2017) United states	To (1) identify skills being performed by advanced practice registered nurses (APRN) practicing in emergency care settings, (2) explore types of training and (3) describe competency validation.	A mixed‐method study. A self‐report survey (57 item) and focus group interviews were conducted to answer the five questions, including: What skills and procedures are used by APRNs in emergency care settings? How often are these skills and procedures being used by APRNs?	Emergency care setting APRNs were recruited from the ENA website (*n* = 146) for the self‐report survey and 24 APRNs participated in focus groups (two groups).	Clinical nurse specialists and nurse practitioners (CNSs and NPs) reported using both direct and indirect care skills in the survey. CNSs reported daily use of direct care skills such as triaging (52%), assessing (12%) and documenting (36%) patient histories, as well as ordering and interpreting electrocardiograms (20%). NPs also reported triaging (87%), assessing (44%), and documenting (89%) patient histories on a daily basis, as well as reporting, assessing and intervening in cases of intimate partner violence (44%), suicidality (33%), and palliative and comfort care (48%); performing skin and wound care procedures (52%); and performing gynaecologic and rectal procedures (45%). In addition, 100% reported ordering and interpreting electrocardiograms on a daily basis. Scope of practice as discussed in the interviews included diagnostic reasoning and patient management, systems management and continuing education need assessments. APRNs made a distinction between overtly clinical hard skills and softer, more intangible clinical reasoning, diagnostic, and systems management skills. Participants seemed to equate more invasive skills with a higher level of practice. Procedures carrying higher risk were mentioned first: intubation, lumbar puncture and chest tube insertion. Skills that are more common, such as suturing and incision and drainage, were perceived to be frequently delegated to nurses. However, there is no standardized scope of practice and APRNs autonomy is affected by physicians, institutional culture, and state boards.	High

Note

Limiters: Publishing date: January 2006‐September 2018, English Language and Peer Reviewed. In the database Scopus studies in press were included.

### 2.6 Analysis and presentation

The integrative data analysis and synthesis were performed as a descriptive thematic analysis inspired by Whittemore and Knafl (2005), allowing data to be grouped according to their findings rather than by method. This descriptive thematic analysis consists of data reduction, data display, data comparison, conclusion and verification. A data extraction form containing the explicit research questions was used for data reduction. These forms were then used to categorize and summarize content related to characteristics and findings in the included studies (Table 3). Data were then analysed (data comparison) by being coded, condensed and then grouped according to patterns and themes. Finally, themes and subthemes were identified and described (data conclusion and verification). Throughout the analysis, comparisons between evolving themes, subthemes and original studies were made to ensure accuracy and reliability.

## 3 RESULTS

A total of 1,343 studies were identified in four databases; 390 studies were duplicates, and as a result of four search and selection rounds (Figure 1), 25 studies were included in the final results, described in Tables 3 and 4. To facilitate reading and traceability, studies connected to findings are indicated by numbers (1–25) in Table 4.

**TABLE 4:** Thematic findings related to respective studies

Studies related to thematic findings Study number^1^, first author and year of publication	Themes							
	Versatile care		Safe care based on nd accuracy		Autonomous performance within boundaries		Beneficial towards patients and society	
	Subthemes							
	First contact ‐ all patient categories	Management of defined patient categories	Assessment, treatment, referral and discharge of patients	Patient safety	Independent service	Blurring boundaries	Patient satisfaction	Cost‐effective and qualitative service
1. Campo et al., (2008)	X	‐	X	‐	X	‐	‐	‐
2. Carlström et al., (2017)	X	X	X	‐	X	‐	X	X
3. Cummins et al., (2013)	X	‐	X	X	X	‐	‐	X
4. Dixon et al., (2009)	X	X	‐	‐	X	X	X	‐
5. Griffin et al., (2016)	X	‐	X	X	X	‐	‐	‐
6. Hui Lau et al., (2012)	‐	X	X	X	X	X	‐	X
7. Jennings et al., (2015)	X	‐	X	‐	X	X	‐	X
8. Jennings et al., (2015)	‐	X	X	‐	X	‐	‐	‐
9. Jennings et al., (2013)	X	‐	X	‐	X	X	X	‐
10. Magnusson et al., (2015)	‐	X	X	X	X	‐	‐	‐
11. Mason et al., (2006)	X	‐	X	‐	‐	‐	‐	X
12. Mason et al., (2008)	‐	X	X	X	X	‐	‐	‐
13. Mc Connel et al., (2013)	‐	‐	X	‐	X	X	‐	‐
14. Mc Devitt et al., (2014)	‐	‐	X	‐	X	X	X	‐
15. Norris et al., (2006)	X	‐	X	‐	‐	X	‐	‐
16. O Keeffe et al., (2011)	‐	X	X	‐	X	‐	‐	X
17. Rubensson Wallin et al., (2018)	‐	X	X	X	X	X	‐	‐
18. Sandhu et al., (2009)	‐	‐	‐	‐	X	‐	‐	‐
19. Snaith et al., (2014)	‐	X	X	X	X	‐	‐	X
20. Swain et al., (2012)	‐	‐	X	‐	X	‐	X	‐
21. Thompson et al., (2012)	X	‐	X	X	X	‐	‐	X
22. van der Linden et al., (2010)	‐	X	X	X	X	‐	‐	X
23. von Vopelius‐Feldt et al., (2013)	‐	X	X	‐	X	‐	‐	X
24. von Vopelius‐Feldt et al., (2014)	X	‐	X	‐	X	X	‐	‐
25. Wolf et al., (2017)	X	‐	X	‐	X	X	‐	‐

### 3.1 Characteristics of the included studies

The review is based on studies from seven countries (United States, United Kingdom, Ireland, Australia, Sweden, New Zealand, and the Netherlands, Table 3), which were published between 2006 and 2018 (md 2013) and included quantitative (*n* = 21) and mixed methods (*n* = 4) studies. Fifteen of the studies focused on in‐hospital emergency care care and 10 focused on pre‐hospital care. Nineteen of them included ANs while the remaining six included APs.

The included studies were assessed as being of high (*n* = 17) and of medium (*n* = 8) quality. Areas assessed to be of some concern in the studies rated as medium were related to: aim^4 5 10 18 23 24^, sample^4 11 18^, data collection^11 18 24^, ethics^10, 11, 23^, results^10 18 24^, strengths and limitations^4 5 10 11 22–24^ and contribution to research^4 5 23 24^. Questions of clarity, relevance, validity and reliability etcetera were included in the respective areas and a lack of clarity and relevance in the study presentation generated a lesser score.

### 3.2 Professional titles in studies and settings

The studies included 13 different professional titles for APs and ANs. One of the studies^11^ focused on ANs in both pre‐hospital and in‐hospital emergency care, while in all other studies APs and ANs were separated into either pre‐hospital or in‐hospital emergency care. In pre‐hospital care, they were given professional titles such as; paramedic practitioner^4 12^, pre‐hospital emergency nurse ^10^, emergency ambulance nurse^2^, critical care paramedic^23 24^, extended care paramedic^20^, emergency care practitioner^11^, advanced paramedic^3^ and pre‐hospital emergency care nurse^17^, and their assignments were related to: ground ambulance^3 11 12 17 20 24^, single responder unit^2 10^, fast responder vehicle^4^, and rapid response vehicle and helicopter^23 24^. Advanced nurses working in emergency departments or minor injury units had professional titles such as; emergency nurse practitioner ^9 13 14 18 19 22^, nurse practitioner^1 6–8^, advanced nurse practitioner^5 21^, emergency care practitioner^11 16^, advanced practice registered nurse^25^ and acute care nurse practitioner^15^. The studies focused on assignments in emergency department care, including fast track and urgent care^1 5–9 11 13 15 18 19 21 22 25^, and minor injury units^13 −16^.

### 3.3 Thematic findings

The descriptive analysis disclosed four themes with two subthemes, respectively (Table 4).

#### 3.3.1 Versatile care

The theme Versatile care reflects on the scope of practice of APs and ANs diverse assignments in different settings related to the management of different patient categories, with different medical needs at different levels of care ^1–12 15–17 19–25^. To some extent, diverse assignments carried out in different settings might also possibly reflect a different focus in advanced level education.

##### First contact – all patient categories

Pre‐hospital and in‐hospital emergency care is characterized by being the first contact of care for patients, and traditionally it means management and care of all patient categories. Management of all patient categories included assessment and treatment of patients of different ages with a variety of illnesses and injuries covering the whole acuity (priority) spectra, from the most critical patients through to non‐critical patients. Fifteen of the studies were related to APs and ANs’ service in pre‐hospital and in‐hospital emergency care, with no specific study aim related to specific patient categories^2‐5 7 9 11 15 21 24 25^.

##### Management of defined patient categories

Eleven studies focused on APs and ANs’ service in relation to defined patient categories. In single responder units, ANs addressed potentially critical patients^1^ and non‐critical patients^10^. APs were studied in relation to older people^4^ suffering minor conditions in the community^12^. Children with minor conditions treated by advanced nurses were focused on in another study^16^. Potentially critical patients were studied; ANs’ assessment and treatment of patients with head trauma^17^ and APs performing pre‐hospital anaesthesia in a critical care team^23^. ANs were studied in relation to patients with minor injuries and illnesses^22^. ANs’ assessment and treatment of foot and ankle injuries were studied in one study^6^ and minor musculoskeletal trauma was studied in another^19^. Pain management prescribed by ANs at the emergency department was studied in yet another study^8^.

#### 3.3.2 Safe care based on precision and accuracy

The theme Safe care based on precision and accuracy reflects on APs and ANs’ diagnostic and decision‐making skills, level‐of‐care decisions and patient safety during the process of care ^1–17 19–25^.

##### Assessment, treatment, referral and discharge of patients

APs and ANs are involved in the entire process of care for some patients, while for others they are more involved in assessment and treatment. APs and ANs made assessments such as patients’ clinical diagnoses and prediction of hospital admission^3^, X‐ray and X‐ray interpretations^6 13 15 19 21 22 25^, pain management^8 21^, performing and interpreting arterial blood gas tests^15^, and echocardiograms^15 25^, to treatments, referrals and discharge^9 11 13 16^. Furthermore, they made decisions regarding level of care^10 12 20^, assessments in accordance with the triage system^10 13^ and clinical guidelines^13^ for head trauma^17^. Three pre‐hospital studies described assessment and treatment related to potential critical patients^17 23 24^. In one of the studies, assessments using ultrasound were described^24^.

Treatments carried out by APs and ANs can possibly be categorized as the management of critical and non‐critical patients. Examples of non‐critical patient treatments in pre‐hospital care (non‐conveyance to hospital) included; dealing with back injuries, tissue injuries, asthma, intoxications^20^ and protocol‐led dispensing of medications such as analgesics, antibiotics and tetanus^12^. Examples of treatment of potentially critical patients included dealing with chest and abdominal pain^20^ and patients with head trauma^17^, organizing rapid sequence induction of anaesthesia, maintaining anaesthesia^23^, creating surgical airways and performing central venous access and thoracotomy^24^. Examples of ANs’ treatment of non‐critical patients at the emergency departments or minor injury units were; taking care of minor injuries and illnesses^5 9 14 21 22^, musculoskeletal trauma^6 19^ and suturing^15^.

A number of specific procedures related to both non‐critical patients and critical patients performed by ANs were described in emergency department context^1 25^. Examples of procedures related to critical patients were adult and child cardiopulmonary arrest, advanced airway management and balloon tamponade for oesophageal varices. Two studies described ANs’ management of patients in relation to the Australasia triage scale, and very few patients were managed in the two most urgent triage categories (critical patients), that is no patients^6^ and one patient (1.6%)^7^.

##### Patient safety

Patient safety relates to APs and ANs service in relation to standard pre‐hospital or in‐hospital emergency care alternatives, and to what extent the outcome of care reflects patient safety. Diagnostic precision was measured in several studies. APs assessed the correct diagnoses in 70% of cases (525/748) and the correct diagnoses were extremely high for issues concerning airway and ventilation, burns, paediatrics and pregnancy emergencies but weaker for allergies and anaphylaxis^3^. ANs’ assessment of patients with fractures and interpretation of X‐rays showed high accuracy (0%–3% missed fractures)^5 6 19 21 22^. APs’ management of older people was found to be as safe as standard pre‐hospital care and transport to emergency department, in relation to re‐presentation at hospital within 7 days or rates of suboptimal care^12^. ANs in a single responder unit assessed 529 patients, 200 of them stayed at the scene or were referred to primary care and 39 patients attended the emergency department within 72 hr with residual symptoms^10^. One pre‐hospital study investigating the assessment and treatment of patients with head trauma indicated suboptimal patient safety^17^, finding that ANs assessed and triaged patients differently according to gender (all core vital signs for women were assessed in 22,9% versus men in 27,2%) and Glasgow Coma Scale was assessed in 30% of the patients.

#### 3.3.3 Autonomous performance within boundaries

The theme Autonomous performance within boundaries reflects APs and ANs’ assignments and performance which to a high degree are characterized by individual and autonomous practice ^1–9 11–25^. However, much of the autonomous practice is conditional to defined categories of patients, assessment and treatment protocols, and they are thereby also affected by the presence of other health care providers.

##### Independent service

In the included studies, APs and ANs either performed autonomously^2 4–6 10 12–14 16 18–22^, with colleagues or in teams^17 23^, or both^1 7–9 24 25^. One study had a design where an APs listened to emergency calls (emergency dispatch centre) to predict hospital admission^3^. APs and ANs in pre‐hospital and emergency department settings autonomously provide emergency health care for different categories of patients.

##### Blurring of boundaries

The scope of practice of APs and ANs are affected by the presence of other providers, institutional culture and regulations of practice. The role of APs and ANs is, to some extent, to fill a gap between the nurses’ and physicians’ service, in order to extend registered nurses’ practice into advanced practice. In some of the reviewed studies, there are examples of APs and ANs being directed to defined patient categories^4 6 7 9 13 14^ and that the service relies on protocols and guidelines^13 17^. The regulators of practice thereby set the boundaries for APs and ANs’ service and uncertainty with these boundaries, between physicians’ skills and roles, was perceived as a barrier towards ANs’ practice in emergency department settings^15^. Protocol use, referral rights and prescribing authority were considered ways that nursing management and medical staff can indirectly control the advanced nurses’ role^13^. Lack of understanding of the ANs’ scope of practice^25^ and lack of evidence of APs’ service^24^ were also perceived barriers for advanced practice.

#### 3.3.4 Beneficial towards patients and society

The theme Beneficial towards patients and society reflects the outcome of APs and ANs’ service, which is perceived as being of high quality and facilitating the care processes with less resource utilization ^2– 4 6– 9 11 14 16 19–23^.

##### Patient satisfaction

There was a high level of patient satisfaction associated with ANs’ service in emergency departments, that is with waiting time, pain management, advice given and communication^4^. Another study measuring waiting time of ANs’ service found that patients with minor injuries in an emergency department setting had to wait 14 min to be seen by an AN^9^. Patients presenting with pain at the emergency department were evaluated and the majority received analgesia from advanced nurses within 30 min ^8^. The vast majority of patients (97%) treated by ANs in a rural urgent care centre perceived that the quality of service was of a high standard^14^. A pre‐hospital single responder unit project was evaluated and findings showed that average waiting time decreased by 49%, from 26 to 13 min during the time of the project^2^. Contrary to other studies, one study's findings indicated that patient satisfaction was not influenced by waiting time^14^. Another study demonstrated that patients were satisfied with the pre‐hospital assessment and treatment by ANs and especially by the avoidance of unnecessary transfers to hospital^20^.

##### Cost effective and qualitative service

Several studies compared APs and ANs’ service with standard care alternatives and if the same results can be achieved with less resources it reflects aspects of cost efficacy. One study compared ANs’ and emergency department doctors assessment of foot injuries using the Ottawa Ankle Rules (requiring X‐ray assessment 78.4% versus ED‐Dr: 88.6% and detection of significant fracture 17.6% versus ED‐Dr: 22.8%) and with almost similar results^6^. Another study compared ANs’ care with standard emergency department care and found no differences between the two groups regarding waiting time (41,5 min versus 39,4 min), length of stay (143,5 min versus 146,7 min), number of patients who left, patient re‐presentations within 48 hr, and the use of evidence‐based guidelines^7^. In studies comparing physician‐led care with AN‐led care, there were no differences in interpretative errors of X‐rays^19 21^ and ANs were found to be equivalent or better in the areas of pain management and reducing waiting time^21^.

No significant differences were found between the ANs and physicians in terms of missed injuries or inappropriate management. Furthermore, there was no significant difference in waiting time for treatment from physicians versus ANs (20 min versus. 19 min). At 85 min, the mean length of stay was significantly longer for physicians; ANs had a mean length of stay of 65 minutes^22^. APs treating older people with minor conditions in the community were as safe as standard pre‐hospital transfers and treatment within the emergency department, in terms of hospital re‐presentation within 7 days or rates of assessed suboptimal care^2^.

Team members in a critical care unit were compared regarding intubation success rate. Successful intubation on the first attempt was achieved in 85% versus. 78% for physicians and APs, respectively^23^. Patient consultation was compared between ANs and physicians and while there was no significant difference in consultation length, ANs and general practitioners (GPs) focused more on educating patients and medical condition or therapeutic regimen than emergency department doctors^16^.

Cost effectiveness was studied, comparing APs with standard pre‐hospital emergency care in older people, and the total costs were found to be lower for APs^4^. When costs and quality‐adjusted life‐year (QALY) were looked at simultaneously, APs’ care had a 95% chance of being cost effective. APs’ management of older patients had lower rates of transport to, attendance at (53% versus. 84%), and time spent in the emergency department (126 min versus. 211 min) than standard pre‐hospital care^3^. However, APs spent more time on scene than standard pre‐hospital care and also increased older patients’ use of health services in the days after the incident. A single responder unit (ANs) project reduced the amount of ambulance calls during the project time by cancelling 33% of the calls, an increase by 12% before the project began^2^. In one study describing pre‐hospital developments, ANs assessed, treated and discharged 43% of their patients^11^. However, in relation to standard care providers treating children with minor conditions, ANs were found to be less effective in terms of discharging fewer patients and referring more patients to hospital or primary care^16^.

## 4 DISCUSSION

Although, the current integrative review found diversities regarding professional titles, roles, scope of practice and available support networks including physical resources, among APs and ANs. The findings also show that these health care professionals have a huge impact on patients’ entire trajectory of care including assessment, treatment, referral and discharge. Patients are to a high degree satisfied with APs and ANs’ care and services. Several studies indicate that patient assessments and treatments were of equal or better quality than standard alternatives. When patients are assessed and treated already at the pre‐hospital stage, unnecessary ambulance transports to emergency departments are avoided, leading to both reduced suffering and wise use of existing resources. The findings described above are congruent with previous studies (Cooper & Grant, 2009; Evans et al., 2013; Sheer & Wong, 2008; Williams, 2017).

APs’ and ANs’ ability to perform autonomously in pre‐hospital and in‐hospital emergency care settings permeated most of the included studies. However, uncertainty and the blurring of boundaries between their professional roles and the roles of other health care providers, such as physicians or managers, were found to restrict the optimal use of their competence. A common understanding of respective professional roles and competence is vital for collaboration. Further research contributing to strengthening and clarifying the evidence base relating to APs’ and ANs’ roles and competence is needed.

An interesting result was shown in the theme Versatile care, which included the challenge of being specialists versus generalists in pre‐hospital and emergency care settings. These contexts demand general and broad competence in contrast to critical care or anaesthetic care where ANs represent advanced specialists (experts in a certain area, described in curriculums and defined by settings and patients). Wilson et al., (2015) claim that pre‐hospital clinicians should be generalists, which logically also translates to emergency department clinicians – as both represent the first contact of patients seeking the respective service. The broad area of expertise needed in pre‐hospital and emergency care might also provide an explanation for the comprehensive use of clinical protocols. A future challenge and important task for APs and ANs and their management is to ensure optimal and flexible use of advanced competence when caring for a variety of patient categories utilizing clinical protocols combined with holistic and person‐centred care.

Effective resource management seems essential for future sustainable health care. In this study, APs and ANs’ service was found to hold the same quality as the alternatives, standard care or physician‐led care, and to be beneficial towards patients and society. Striving for sustainability in pre‐hospital and in‐hospital emergency care includes the optimal use of APs and ANs competence, and a self‐evident strategy is therefore to direct assignments to the profession that can complete it at the lowest total cost with retained quality (SOU:, 2016:2).

The results in this study, with some exceptions, lacked examples of how APs and ANs contributed to advanced care in critical situations. Time critical pre‐hospital care outcomes, to some extent, depend on the ability to “do the right things early on” (Wilson et al., 2015) and physician‐led pre‐hospital service is limited and cannot always be expected to be available. Aspects of core competencies (Cronewett et al., 2009), the holistic perspective on competence (Black et al., 2008; Cowan et al., 2007) and the domains of advanced nurse practice (Dowling et al., 2013; Jokiniemi et al., 2012; Mantzoukas & Watkinson, 2007) were hardly mentioned in the studies included in this review. However, aspects of these competencies are used in clinical encounters and all of the included studies had an overall clinical focus. With studies exploring advanced practice by APs and ANs in relation to critical patients and to all advanced practice domains, further motivation can be achieved for utilization of advanced competence.

### 4.1 Methodological considerations

Using wide inclusion criteria for population and setting has given this review the desired richness and variation of examples. The decision was based on the expected limited amount of studies of APs and ANs in pre‐hospital care. The inclusion criteria of paramedics and nurses with advanced level of education were a strategy to minimize the risk of missing relevant professional titles, which worked out to some degree, but also led to the exclusion of some articles during the selection process (primarily due to the inability to find a formal advanced level of education in populations).

The quality of the selection of studies and data evaluations was considered a strength. At each step of the selection process, quality assessment and data extraction, all three authors were involved in discussions until consensus was reached. The modified critical appraisal tool used included a numeric scoring system that allowed individual assessment and facilitated continuity in the assessments. In 21 studies, the level of quality was agreed on already at an individual level. All included studies were discussed at a consensus meeting and their quality assessment was agreed on. However, using another critical appraisal tool might have rated the studies differently.

Conducting this integrative review focusing on APs and ANs’ practice within pre‐hospital and in‐hospital emergency care settings has not been carried out without challenges. The biggest challenges were to comprehend the phenomena of interest and to summarize and display the findings. The vast amount of professional titles, generated from different advanced level education programmes with different curricula and goals, was evident in the present review. To manage all the professional titles, the included populations were referred to as APs and ANs, which may have implications for understanding, reliability and validity. To compensate for that, numeric referencing of studies has been used for transparency and traceability in the result section, see Tables 3 and 4.

With a more comprehensive understanding of the heterogeneity of professional titles, the search terms used could have been improved to further strengthen the study. Olaussen et al., (2017) discussed the complexity of finding relevant search terms relating to paramedics and also created two search filters, which would possibly enabled optimized sensitivity and specificity in database search, in this study. An improvement of search terms appears to be best motivated in relation to the “outcome” related terms, that is paramedic role, scope of practice, further variations of competence, skills and to the domains of advanced nursing practice (Dowling et al., 2013; Jokiniemi et al., 2012). Possibly, qualitative studies could also have been included with improved search terms.

It was challenging to synthesize the findings, due to the studies different methodological approaches, variables and detail levels. With such a broad phenomenon of interest, specificity may be lost in the synthesis of findings, which was a calculated risk motivated by the possibility of contributing to future policy making, competence strategies and future research. Consequently, this synthesis ended in an attempt to describe, count, compare and cluster, noting patterns and themes from findings with different abstraction and detail levels (Whittenmore & Knafl, 2005).

## 5 CONCLUSION

The findings showed various examples of interventions performed by APs and ANs, in different roles, context and to defined or undefined patient categories. The care and service performed by APs and ANs in pre‐hospital and in‐hospital emergency care has the potential to be safe, of high quality, and of public benefit. However, their roles and functions may be further established and extended by exploring the boundaries between theirs and physicians’ scope of practice, and the domains included in the concept of “advanced practice.”

## 6 COMPETING INTERESTS

The authors declare that they have no competing interests.

## AUTHORS' CONTRIBUTIONS

The authors designed the study together. JJ performed the literature search and all three authors actively took part in the selection process. JJ drafted the manuscript and JN and ML gave advice and made critical revisions throughout the process of conducting this study.

## Data Availability

The authors confirm that the data supporting the findings of this integrative review are available within the articles in the result section, indicated by numbers, in the Tables 3 and 4 and in the references.

## References

[nop2866-bib-0001] Aiken, L. H., Sloane, D. M., Bruyneel, L., Van den Heede, K., Griffiths, P., Busse, R., Diomidous, M., Kinnunen, J., Kózka, M., Lesaffre, E., McHugh, M. D., Moreno‐Casbas, M. T., Rafferty, A. M., Schwendimann, R., Scott, P. A., Tishelman, C., van Achterberg, T., & Sermeus, W. (2014). Nurse staffing and education and hospital mortality in nine European countries: A retrospective observational study. Lancet, 383(9931), 1824–1830. 10.1016/S0140-6736(13)62631-8 24581683 PMC4035380

[nop2866-bib-0002] Al‐Shaqsi, S. (2010). Models of international emergency medical service (EMS) system. Oman Medical Journal, 25(4), 320–323. 10.5001/omj.2010.92 22043368 PMC3191661

[nop2866-bib-0003] Begley, C., Elliot, N., Lalor, J., Couyne, I., Higgins, A., & Comiskey, C. (2013). Differences between clinical specialists and advanced practitioner clinical practice, leadership, and research roles, responsibilities, and perceived outcomes (the SCAPE study). Journal of Advanced Nursing, 69(6), 1323–1337. 10.1111/j.1365-2648.2012.06124.x 22931391

[nop2866-bib-0004] Black, J., Allen, D., Redfern, L., Muzio, L., Rushowick, B., Balaski, B., Martens, P., Crawford, M., Conlin‐Saindon, K., Chapman, L., Gautreau, G., Brennan, M., Gosbee, B., Kelly, C., & Round, B. (2008). Competencies in the context of entry‐level registered nurse practice: A collaborative project in Canada. International Nursing Review, 55, 171–178. 10.1111/j.1466-7657.2007.00626.x 18477101

[nop2866-bib-0005] Campo, T., McNulty, R., Sabatini, M., & Fitzpatrick, J. (2008). ^1^. Nurse practitioners performing procedures with confidence and independence in the emergency care setting. Advanced Emergency. Nursing Journal, 30(2), 153–170. 10.1097/01.TME.0000319926.97473.8d

[nop2866-bib-0006] Canadian Nurses Association . (2008). Advanced nursing practice: a national framework. www.cna‐aiic.ca

[nop2866-bib-0007] Carlström, E., & Fredén, L. (2017). ^2^. The first single responders in Sweden – evaluation of a prehospital single staffed unit. International Emergency Nursing, 32, 15–19. 10.1016/j.ienj.2016.05.003 27282963

[nop2866-bib-0008] College of Paramedics (2018). The college of paramedics. Post registration ‐ Paramedic career framework 4th Edition. www.collegeofparamedics.co.uk ISBN 978‐0‐9558429‐9‐3.

[nop2866-bib-0009] Cooper, J. R., & Grant, J. (2009). New and emerging roles in out of hospital emergency care: A literature review. International Emergency Nursing, 17, 90–98. 10.1016/j.ienj.2008.11.004 19341994

[nop2866-bib-0010] Cowan, D. T., Wilson‐Barnett, J., & Norman, I. J. (2007). A European survey of general nurses self assessment of competence. Nurse Education Today, 27(5), 452–458. 10.1016/j.nedt.2006.08.008 17097196

[nop2866-bib-0011] Cronewett, L., Sherwood, G., Pohl, J., Barnsteiner, J., Moore, S., Sullivan, D. T., Ward, D., & Warren, J. (2009). Quality and safety education for advanced nursing practice. Nursing Outlook, 57(6), 338–348. 10.1016/j.outlook.2009.07.009 19942035

[nop2866-bib-0012] Cummins, N. M., Dixon, M., Garavan, C., Landymore, E., Mulligan, N., & O'Donnell, C. (2013). ^3^. Can advanced paramedics in the field diagnose patients and predict hospital admission? Emergency Medical Journal, 1–5. 10.1136/emermed-2012-201899 23407377

[nop2866-bib-0013] Dixon, S., Mason, S., Knowles, B., Colwell, B., Wardrope, J., Snooks, H., Gorringe, R., Perrin, J., & Nicholl, J. (2009). ^4^. Is it cost effective to introduce paramedic practitioners for older people to the ambulance service? Results of a cluster randomised controlled trial. Emergency Medical Journal, 26, 446–451. 10.1136/emj.2008.061424 19465624

[nop2866-bib-0014] Dowling, M., Beauchesne, M., Farrelly, F., & Murphy, K. (2013). Advanced practice nursing: A concept analysis. International Journal of Nursing Practice, 19(2), 131–140. 10.1111/ijn.12050 23577970

[nop2866-bib-0015] Ebben, R. H. A., Vloet, L. C. M., Verhofstad, M. H. J., Meijer, S., Mintjes‐de Groot, J. A. J., & van Achterberg, T. (2013). Adherence to guidelines and protocols in the prehospital and emergency care setting: A systematic review. Scandinavian Journal of Trauma, Resuscitation and Emergency Medicine, 21, 9. 10.1186/1757-7241-21-9 PMC3599067 23422062

[nop2866-bib-0016] Evans, E., McGovern, R., Birch, J., & Newbury‐Birch, D. (2013). Which extended paramedic skills are making an impact in emergency care and can be related to the UK paramedic system? A systematic review of the literature. Emergency Medical Journal, 1–10. 10.1136/emermed-2012-202129 PMC4078671 23576227

[nop2866-bib-0017] Farrohknia, N., Castrén, M., Ehrenberg, A., Lind, L., Oredsson, S., Jonsson, H., Asplund, K., & Göransson, K. E. (2011). Emergency department triage scales and their components: A systematic review of the scientific evidence. Scandinavian Journal of Trauma, Resuscitation and Emergency Medicine, 19(1), 42– 10.1186/1757-7241-19-42 PMC3150303 21718476

[nop2866-bib-0018] Gardett, I., Clawson, J., Scott, G., Barron, T., Patterson, B., & Olola, C. (2013). Past, Present, and Future of Emergency Dispatch Research: A Systematic Literature Review. Annals of Emergency Dispatch & Response, 1(2), 29–42.

[nop2866-bib-0019] Gardner, G., Chang, A., & Duffield, C. (2007). Making nursing work: Breaking through the role confusion of advanced practice nursing. Journal of Advanced Nursing, 57(4), 382–391. 10.1111/j.1365-2648.2007.04114.x 17291202

[nop2866-bib-0020] Griffin, M., & Joe McDevitt, J. (2016)^5^. An evaluation of the quality and patient satisfaction with an advanced nurse practitioner service in the emergency department. The Journal for Nurse Practitioners, 12(8), 553–559.

[nop2866-bib-0021] Hoot, N. R., & Aronsky, D. (2008). Systematic Review of Emergency Department Crowding: Causes, Effects, and Solutions. Annals of Emergency Medicine, 52(2), 126–136. 10.1016/j.annemergmed.2008.03.014 18433933 PMC7340358

[nop2866-bib-0022] Hui Lau, L., Kerr, D., Law, I., & Ritchie, P. (2013). ^6^. Nurse practitioners treating ankle and foot injuries using the Ottawa Ankle Rules: A comparative study in the emergency department. Australasian Emergency Nursing Journal, 16, 110–115. 10.1016/j.aenj.2013.05.007 23953094

[nop2866-bib-0023] International Council of Nurses . (2016). International Nurse Practitioner/Advanced Practice Nursing Network: Definition and Characteristics of the role. https://international.aanp.org/Practice/APNRoles

[nop2866-bib-0024] Jennings, N., Gardner, G., O’Reilly, G., & Mitra, B. (2015). ^7^. Emergency NP model of care in an Australian emergency department. The Journal for Nurse Practitioners, 11(8), 774–781. 10.1016/j.nurpra.2015.05.008

[nop2866-bib-0025] Jennings, N., Kansal, A., O’Reilly, G., Mitra, B., & Gardner, G. (2015). ^8^. Time to analgesia for care delivered by nurse practitioners in the emergency department ‐ a retrospective chart audit. International Emergency Nursing, 23, 71–74. 10.1016/j.ienj.2014.07.002 25113664

[nop2866-bib-0026] Jennings, N., Mckeown, E., O’Reilly, G., & Gardner, G. (2013). ^9^. Evaluating patient presentations for care delivered by emergency nurse practitioners: A retrospective analysis of 12 months. Australasian Emergency Nursing Journal, 16, 89–95. 10.1016/j.aenj.2013.05.005 23953091

[nop2866-bib-0027] Jokiniemi, K., Pietilä, A.‐M., Kylmä, J., & Haatainen, K. (2012). Advanced nursing roles: A systematic review. Nursing and Health Sciences, 14, 421–431. 10.1111/j.1442-2018.2012.00704.x 22950621

[nop2866-bib-0028] Khorram‐Manesh, A., Lenquist Montán, K., Hedelin, A., Kihlgren, M., & Örtenwall, P. (2011). Prehospital triage, discrepancy in priority‐setting between emergency medical dispatch centre and ambulance crews. European Journal of Trauma Emergency Surgeons, 37, 73–78. 10.1007/s00068-010-0022-0 26814754

[nop2866-bib-0029] Lowthian, J. L., Cameron, P. A., Stoelwinder, J. U., Curtis, A., Currel, A., Cooke, M. W., & McNeil, J. J. (2011). Increasing utilization of emergency ambulances. Australian Health Review, 35, 63–69. 10.1071/AH09866 21367333

[nop2866-bib-0030] Lowthian, J. A., Jolley, D. J., Curtis, A. J., Currell, A., Cameron, P. A., Stoelwinder, J. U., & McNeil, J. J. (2011). The challenges of population ageing: Accelerating demand for emergency ambulance services by older patients, 1995–2015. MJA (Rapid Online Publication), 194(11), 574–578. 10.5694/j.1326-5377.2011.tb03107.x 21644869

[nop2866-bib-0031] Magnusson, C., Källenius, C., Knutsson, S., Herlitz, J., & Axelsson, C. (2015). ^10^. Pre‐hospital assessment by a single responder: The Swedish ambulance nurse in a new role: A pilot study. International Emergency Nursing, 26, 32–37. 10.1016/j.ienj.2015.09.001 26472522

[nop2866-bib-0032] Mantzoukas, S., & Watkinson, S. (2007). Review of advanced nursing practice: The international literature and developing the generic features. Journal of Clinical Nursing, 16, 28–37. 10.1111/j.1365-2702.2006.01669.x 17181664

[nop2866-bib-0033] Mason, S., Coleman, P., O’Keeffe, C., Ratcliffe, R., & Nicholl, J. (2006). ^11^. The evolution of the emergency care practitioner role in England: Experiences and impact. Emergency Medical Journal, 23, 435–439. 10.1136/emj.2005.027300 PMC2564336 16714501

[nop2866-bib-0034] Mason, S., Knowles, E., Freeman, J., & Snooks, H. (2008). ^12^. Safety of paramedics with extended skills. Academic Emergency Medicine 15, 607–612. Emergency Medicine Australasia, 24, 175–180. 10.1111/j.1553-2712.2008.00156.x 18691211

[nop2866-bib-0035] McConnell, D., Slevin, O. D., & McIlfatrick, S. J. (2013). ^13^. Emergency nurse practitioners perceptions of their role and scope of practice: Is it advanced practice? International Emergency Nursing, 21, 76–83. 10.1016/j.ienj.2012.03.004 23615513

[nop2866-bib-0036] McDevitt, J., & Melby, V. (2014). ^14^. An evaluation of the quality of Emergency Nurse Practitioner services for patients presenting with minor injuries to one rural urgent care centre in the UK: A descriptive study. Journal of Clinical Nursing, 24, 523–535. 10.1111/jocn.12639 24891126

[nop2866-bib-0037] Moher, D., Liberati, A., Tetzlaff, J., & Altman, D. G. (2009). The PRISMA Group. Preferred Reporting Items for Systematic Reviews and Meta‐Analyses: The PRISMA Statement. PLoS Med, 6(7), e1000097. 10.1371/journal.pmed1000097 19621072 PMC2707599

[nop2866-bib-0038] Norris, T., & Melby, V. (2006). ^15^. The Acute Care Nurse Practitioner: Challenging existing boundaries of emergency nurses in the United Kingdom. Journal of Clinical Nursing, 15, 253–263. 10.1111/j.1365-2702.2006.01306.x 16466474

[nop2866-bib-0039] O’Keeffe, C., Mason, S., Bradburn, M., & Iheozor‐Ejiofor, Z. (2011). A community intervention trial to evaluate emergency care practitioners in the management of children. Archives of Disease in Childhood, 96, 658–663. 10.1136/adc.2010.201889 21505224

[nop2866-bib-0040] Olaussen, A., Semple, W., Oteir, A., Todd, P., & Williams, B. (2017). Paramedic literature search filters: optimised for clinicians and academics. BMC Medical Informatics and Decision Making, 17(1), 1–6. 10.1186/s12911-017-0544-z 29020951 PMC5637081

[nop2866-bib-0041] Public Health Resources Unit . (2006). Critical Appraisal Skills Programme (CASP): making sense of evidence. http://www.casp‐uk.net/

[nop2866-bib-0042] Rantala, A., Behm, L., & Rosén, H. (2019). Quality is in the eye of the beholder – A focus group study from the perspective of ambulance clinicians, physicians, and managers. Healthcare, 7(41), 1–13. 10.3390/healthcare7010041 PMC6473421 30871138

[nop2866-bib-0043] Robertson‐Steel, I. (2006). Evolution of triage systems. Emergency Medical Journal, 23, 154–155. 10.1136/emj.2005.030270 PMC2564046 16439754

[nop2866-bib-0044] Rubenson Wahlin, R., Lindström, V., Ponzer, S., & Vicente, V. (2018). ^17^. Patients with head trauma: A study on initial prehospital assessment and care. International Emergency Nursing, 36, 51–55. 10.1016/j.ienj.2017.10.001 29191378

[nop2866-bib-0045] Sandhu, H., Dale, J., Stallard, N., Crouch, R., & Glucksman, E. (2009). ^18^. Emergency nurse practitioners and doctors consulting with patients in an emergency department: A comparison of communication skills and satisfaction. Emergency Medical Journal, 26, 400–404. 10.1136/emj.2008.058917 19465607

[nop2866-bib-0046] Sandsdalen, T., Hov, R., Höye, S., Rystedt, I., & Wilde‐Larsson, B. (2015). Patients preferences in palliative care: A systematic mixed studies review. Palliative Medicine, 29(5), 399–419. 10.1177/0269216314557882 25680380

[nop2866-bib-0048] Sheer, B., & Wong, F. K. Y. (2008). The development of advanced nursing practice globally. Journal of Nursing Scholarship, 40(3), 204–211. 10.1111/j.1547-5069.2008.00242.x 18840202

[nop2866-bib-0049] Snaith, B., & Hardy, M. (2014). ^19^. Emergency department image interpretation accuracy: The influence of immediate reporting by radiology. International Emergency Nursing, 22, 63–68. 10.1016/j.ienj.2013.04.004 23726985

[nop2866-bib-0050] SOU . (2016). 2. Effektiv vård. Statens offentliga utredningar. (Effective care. The governments official investigations).

[nop2866-bib-0051] Stasa, H., Cashin, A., Buckley, T., & Donoghue, J. (2014). Advancing advanced practice – Clarifying the conceptual confusion. Nurse Education Today, 34, 356–361. 10.1016/j.nedt.2013.07.012 23953150

[nop2866-bib-0052] Swain, A. H., Al‐Salami, M., Hoyle, S. R., & Larsen, P. D. (2012). ^20^. Patient satisfaction and outcome using emergency care practitioners in New Zealand. Emergency Medicine Australasia, 24(2), 175–180. 10.1111/j.1742-6723.2011.01525.x 22487667

[nop2866-bib-0053] Thompson, W., & Meskell, P. (2012). ^21^. Evaluation of an Advanced Nurse Practitioner (Emergency Care) ‐ An Irish Perspective. The Journal for Nurse Practitioners, 8(3), 200–205. 10.1016/j.nurpra.2011.09.002

[nop2866-bib-0054] Tohira, H., Williams, T. A., Jacobs, I., Bremmer, A., & Finn, J. (2014). The impact of new prehospital practitioners on ambulance transportation to the emergency department: A systematic review and meta‐analysis. Emergency Medical Journal, 31, 88–94. 10.1136/emermed-2013-202976 24243486

[nop2866-bib-0055] van der Linden, C., Reijnen, R., & de Vos, R. (2010). ^22^. Diagnostic accuracy of emergency nurse practitioners versus physicians related to minor illnesses and injuries. Journal of Emergency Nursing, 36(4), 311–316. 10.1016/j.jen.2009.08.012 20624563

[nop2866-bib-0056] Von Vopelius‐Feldt, J., & Benger, J. (2013). ^23^. Prehospital anaesthesia by physician and paramedic critical care team in Southwest England. European Journal of Emergency Medicine, 20, 382–386. 10.1097/MEJ.0b013e32835b08b7 23117421

[nop2866-bib-0057] Von Vopelius‐Feldt, J., & Benger, J. (2014). ^24^. Critical care paramedics in England: A national survey of ambulance services. European Journal of Emergency Medicine, 21(4), 301–304. 10.1097/MEJ.0000000000000085 24076658

[nop2866-bib-0058] Whittemore, R., & Knafl, K. (2005). The integrative review: Updated methodology. Journal of Advanced Nursing, 52(5), 546–553. 10.1111/j.1365-2648.2005.03621.x 16268861

[nop2866-bib-0059] Wihlborg, J. (2018). The ambulance nurse. Aspects on competence and education. Lund University: Faculty of Medicine Doctoral Dissertation Series.

[nop2866-bib-0060] Williams, K. (2017). Advanced practitioners in emergency care: A literature review. Emergency Nurse, 25(4), 36–41. 10.7748/en.2017.e1685 28703051

[nop2866-bib-0061] Wilson, M. H., Habig, K., Wright, C., Hughes, A., Davies, G., & Imray, C. H. E. (2015). Pre‐hospital emergency medicine. Lancet, 386, 2526–2534. 10.1016/S0140-6736(15)00985-X 26738719

[nop2866-bib-0062] Wolf, L. A., Delao, A. M., Perhats, C., Moon, M. D., & Carman, M. J. (2017). ^25^. The experience of advanced practice nurses in US emergency care settings. Journal of Emergency Nursing, 43(5), 426–434. 10.1016/j.jen.2017.04.007 28579285

